# Comparison of Early Oral Feeding With Traditional Oral Feeding After Total Gastrectomy for Gastric Cancer: A Propensity Score Matching Analysis

**DOI:** 10.3389/fonc.2019.01194

**Published:** 2019-11-07

**Authors:** Juan Wang, Min Yang, Quan Wang, Gang Ji

**Affiliations:** ^1^Department of Digestive Surgery, Xi Jing Hospital, The Fourth Military Medical University, Xi'an, China; ^2^Emergency Department, Huangpu Branch of the Ninth People's Hospital Affiliated to the Medical College of Shanghai Jiaotong University, Shanghai, China

**Keywords:** early oral feeding, traditional oral feeding, total gastrectomy, gastric cancer, propensity score matching

## Abstract

**Background:** The present study aimed to compare the feasibility and safety of early oral feeding (EOF) with traditional oral feeding (TOF) after radical total gastrectomy for gastric cancer.

**Methods:** This retrospective study included consecutive patients who underwent total gastrectomy from April 2016 and November 2018. These patients were divided into two groups, according to their postoperative feeding protocol: EOF group (*n* = 314) and TOF group (*n* = 433). Propensity score matching was used to balance the potential confounders, and 276 patients were selected from each group. The EOF group received oral diet on postoperative day one, while the TOF group were started on oral feeding after the passage of flatus.

**Results:** No significant differences were found in the postoperative complications (*P* = 0.426) and tolerance to oral feeding (*P* > 0.056) between the two groups. The changes in perioperative nutritional markers were also similar between the two groups (*P* > 0.05). The time to first passage of flatus or defecation (47.19 ± 12.00 h vs. 58.19 ± 9.89 h, *P* < 0.0001) and length of postoperative hospital stay (6.84 ± 2.31 days vs. 7.72 ± 2.86 days, *P* < 0.0001) were significantly lower in the EOF group compared to the TOF group.

**Conclusion:** EOF may be safe and feasible after radical total gastrectomy with faster recovery and no increased risk of postoperative complications.

## Introduction

Gastric cancer is the third most common cause of cancer-related death, and has the fifth highest incidence of cancer worldwide (1). Most of these patients require multimodality treatment including endoscopic resection, surgical resection, chemotherapy, immunotherapy, and radiation therapy. The stomach is also the most common site for gastrointestinal lymphoma (2). However, the treatment for both these diseases are completely different. For early stage gastric adenocarcinoma, complete endoscopic, or surgical resection is the only curative treatment as recommended by the NCCN guidelines (3). Chemotherapy and immunotherapy are used in the neoadjuvant and adjuvant settings to take care of the micro-metastases and cannot achieve complete pathological response. Hence, they are not the preferred first line therapies. Unlike adenocarcinoma, gastric lymphoma shows very good response to chemotherapy, and Helicobacter pylori eradication therapy. Hence, chemotherapy is the first-line treatment for gastric lymphoma (4).

Traditionally, oral feeding is delayed after gastric surgery, until the passage of flatus, or appearance of audible bowel sounds or bowel movements due to the theoretical concerns of increased risk of anastomosis leakage (5). The rationale of nil by mouth was to allow the anastomosis to heal before being stressed by food (6). However, recent studies have questioned this traditional concept of fasting until passage of flatus after gastric surgery (7–9).

Contrary to the widespread belief, various studies have confirmed the safety and feasibility of early oral feeding (EOF) (6, 7, 10–13). A randomized clinical trial performed by Hur et al. demonstrated that EOF was feasible, and could result in shorter hospitalization and improvements in the quality of life of 54 patients receiving open gastrectomy (7). A case-control study and pilot study revealed that EOF after gastrectomy is feasible, with no increase in morbidity (8, 14). A meta-analysis of patients who underwent distal gastrectomy also revealed that EOF is safe and feasible (15). Another systematic review and meta-analysis conducted by Liu et al. revealed that EOF after gastrectomy did not increase the incidence of postoperative complications or readmissions, and significantly reduced the length of hospital stay (15). Furthermore, a recent meta-analysis of six randomized controlled trials (RCTs) of gastric cancer surgery (15) and the combined meta-analysis of 15 RCTs and other studies of open upper gastrointestinal surgery (16) revealed that EOF could reduce the length of hospital stay and bowel recovery time without increasing postoperative complications in gastrectomy patients. Moreover, early oral nutrition is one of the most important elements of the enhanced recovery after surgery (ERAS) strategy after gastrointestinal surgery (7, 8).

Although a number of studies have reported the outcomes of EOF after gastric surgery, most of these studies have focused on EOF after distal gastrectomy. Hence, the outcomes of early oral nutrition after total gastrectomy for gastric cancer is scarce and limited. In recent years, gastric cancer surgery has developed from open surgery, laparoscopic-assisted surgery, to total laparoscopic surgery. However, due to the high position of the gastroesophageal junction and small operating space, it remains difficult to laparoscopically perform a complete total gastrectomy. At present, in China, pure laparoscopic total gastrectomy is only performed in few high-volume centers, and its safety and long-term survival effects have not been confirmed by large-scale clinical studies. Therefore, in the present study, a single center retrospective cohort study was conducted using propensity score matching, in order to compare EOF with traditional oral feeding (TOF) following total gastrectomy (for both open and laparoscopic surgery) in gastric cancer patients. The propensity score matching analysis was used to for the confounding factors by forming a matched cohort, taking in to consideration various variables (17).

## Patients and Methods

### Study Design and Propensity Score Matching

**Figure 1:**
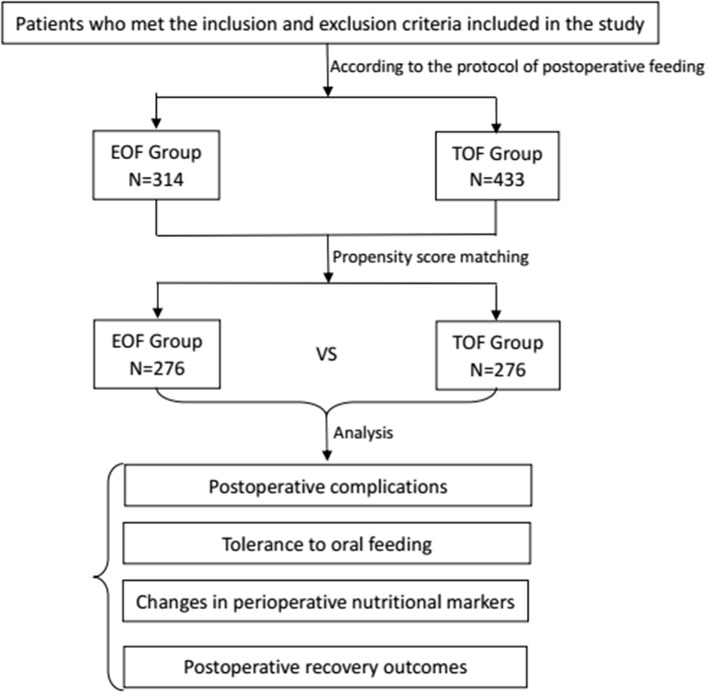
Flow chart depicting the patient selection, propensity score matching, and postoperative variables used for analysis (EOF, early oral feeding; TOF, traditional oral feeding).

In the present study, the data of patients who underwent radical total gastrectomy for gastric carcinoma between April 2016 and November 2018 at the Department of Gastrointestinal Surgery of Xijing Hospital Affiliated to the Fourth Military Medical University were retrospectively analyzed (Figure 1). According to the time when oral nutrition was initiated, these patients were divided into two groups: EOF group and TOF group. Propensity score matching was performed using the logistic regression model to generate a propensity score for balancing the baseline characteristics and potential confounders between these two groups. Specifically, the tendency score was used to synthesize all the observed variable information and balance the variables in order to reduce bias. In the present study, patients were matched one-to-one by propensity score (random selection from severe nearest-neighbor individual propensity score match of which difference between the standard and the matching value <0.001, calculated and matched with logistic regression, a caliper of 0.2 without replacement) using the covariates of age, gender, body mass index (BMI), NRS2002 score (18), American Society of Anesthesiologists (ASA) score, tumor differentiation, clinical stage, and surgical approach as the confounding variables for propensity score matching. The propensity scoring function of the SPSS 22.0 software was used to perform the variable matching. The confounding factors were balanced in the two groups after the propensity score matching. McNemar's Test was used for sensitivity analysis (19). Then, the matched and adjusted cohort data were analyzed, and the results were obtained.

NRS2002 was first introduced by Kondrup (18), and has three components: impaired nutritional status (0–3 points), severity of disease (0–3 points), and age (0–1 points). Patients with NRS 2002 <3 and ≥3 (original scale) were classified as “no nutritional risk” and “nutritional risk,” respectively.

### Selection Criteria

The present study included patients with histologically proven gastric adenocarcinoma by preoperative gastroscopy biopsy or postoperative pathology, who received radical total gastrectomy with R0 resection margins.

The exclusion criteria were as follows: (1) patients with severe cardiovascular disease, respiratory disease, hepatopathy, renal impairment, and abnormal nutritional status; (2) patients with metastatic disease or another type of cancer; (3) patients with a history of neoadjuvant chemo/radiotherapy; (4) patients who underwent emergency operation due to gastric perforation or bleeding; (5) patients with serious complications such as major bleeding occurring within 24 h after surgery, which may affect the oral feeding; (6) patients with combined resection of other organs (except for the gallbladder) or thoracotomy; (7) patients with a preoperative NRS2002 score >3 points or BMI <17 kg/m^2^; (8) patients who were admitted to the intensive care unit (ICU) after surgery.

An informed consent was obtained from all patients. The Institutional Review Board of the Air force Military Medical University approved the present study.

### Postoperative Feeding

For patients in the EOF group, oral feeding was initiated by giving water on the first postoperative day. These patients were started on a clear liquid diet on the second postoperative day, which contained glucose, sodium chloride water, and enteral nutrient solution. From the third postoperative day up to the day of discharge, patients were instructed to eat a liquid diet, and when they passed the flatus or bowel sounds appeared, soft diet was gradually given. The daily calorie requirements were met by supplementing with parenteral nutrition (1,200–1,400 kcal, 20–25 mL/kg/d). For patients who developed intractable nausea, vomiting, or distension, the diet was stopped, and a nasogastric tube was inserted.

For patients in the TOF group, oral feeding was started by giving water when the bowel sounds were audible, or with the passage of flatus. Prior to that, patients were maintained nil-by-mouth, and the daily calorie requirements were provided by parenteral nutrition. A clear liquid diet was given on the next day, and a soft diet was gradually given when the liquid diet was well-tolerated. The diet was stopped and a nasogastric tube was inserted when patients complained of intractable nausea, vomiting, or abdominal distension.

### Perioperative Treatment

Before surgery, preoperative bowel preparation was avoided. Both groups received similar prophylactic antibiotics before the skin incision. General anesthesia with similar anesthetic drugs was administered to all patients. All surgeries were performed by experienced surgeons. Total gastrectomy with a Roux-en-Y esophagojejunostomy reconstruction and D2 lymph node dissection was performed for all patients, as described in the Japanese gastric cancer treatment guidelines (20). All anastomoses were strengthened by a 3–0 silk thread. A nasogastric tube and urinary catheter were routinely inserted in the operating room, and was removed on the morning of postoperative day (POD) 1. An abdominal drain was also routinely placed. Postoperative pain was managed by non-steroidal anti-inflammatory drugs (NSAIDs), but no epidural analgesia was given. All the patients were encouraged to start active ambulation from POD1. Patients in both the groups were discharged using the same criteria. The criteria for discharge were as follows: (1) no pyrexia; (2) passage of flatus and feces in the postoperative period; (3) removal of the abdominal drain; (4) no obvious symptoms such as nausea, vomiting and abdominal distention; (5) tolerance of oral feeding for at least 24 h. When the patients were suspected to have anastomotic complications, such as anastomotic leakage or ileus, oral intake was immediately stopped, and the appropriate treatment was given.

### Data Collection and Definitions

Data regarding the demographic and clinicopathologic characteristics of the patients were retrieved from the medical records. The following data was collected: age, gender, NRS2002 score, ASA score, BMI, histologic differentiation, pathological stage, surgical approach (laparoscopic surgery or open surgery), complications (including anastomotic leakage, duodenal stump leakage, pancreatic fistula, abdominal bleeding, abdominal infection, pulmonary infection, wound infection, wound dehiscence, and ileus), the prevalence and grade of all postoperative complications (revised Clavien-Dindo classification) (21), reoperation, re-hospitalization, 30-day mortality, oral feeding tolerance (including postoperative nausea or vomiting, and abdominal distention), changes in nutritional markers (preoperative and postoperative serum albumin and serum prealbumin level on POD1 and POD3), time to first passage of flatus or feces, and length of postoperative hospital stay.

The pathological stage was performed according to the seventh edition of the American Joint Committee on Cancer TNM classification of gastric carcinoma. Complications were detected using the clinical symptoms, or the laboratory and imaging tests. The anastomotic leakage was radiologically (extravasation of the contrast medium at the anastomotic site) or clinically (abdominal pain, fever, and discharge of gastrointestinal content through a drain) confirmed. Postoperative morbidity and mortality were defined as complications or deaths within 30 days after surgery.

### Follow-Up

After discharge from the hospital, patients were followed up in the Outpatient Department or by telephone. Blood or imaging studies (X-radiography, CT scan, or angiography) were performed according to the clinical symptoms.

### Statistical Analysis

Data were expressed as mean ± standard deviation (SD), or number and percentage. The categorical variables were compared using chi-square test, and Student *t*-test was used to compare continuous variables. The difference in changes in postoperative nutritional markers was analyzed by two-way repeated-measures ANOVA. Two-sided *P*-values <0.05 were considered to be statistically significant. The statistical analysis was performed using the IBM® SPSS® Statistics Version 22.0 (Corp, Armonk, NY, USA).

## Results

### Patient Characteristics Before and After Propensity Score Matching

A total of 747 patients were included in the present study. Among these patients, 314 patients (42.03%) were treated with EOF, while the remaining 433 patients (57.97%) received TOF. The clinicopathological characteristics of these patients are presented in Table 1. Before the propensity score matching, there were significant differences in gender (*P* = 0.022), histological differentiation (*P* < 0.0001), and surgical approach (*P* < 0.0001) between the EOF and TOF groups (Table 1). However, there were no statistically significant differences in age, NRS2002 score, ASA score, BMI and pathological stage between the two groups (Table 1). After the propensity score matching, 276 patients were selected from each group, and the baseline characteristics were well-balanced between the two matched groups (Table 1).

**Table 1:** Patient demographics and baseline clinicopathological characteristics before and after propensity score matching.

**Characteristics**	**Before matching**		**χ^**2**^**	**P1 value**	**After matching**		**χ^**2**^**	**P2 value**
	**EOF group**	**TOF group**			**EOF group**	**TOF group**		
Age (years)			0.343	0.558			0.000	1.000
≤60	162	214			140	140		
>60	152	219			136	136		
Gender			5.246	0.022			0.011	0.915
Male	244	365			222	221		
Female	70	68			54	55		
NRS2002 Score			0.727	0.394			0.034	0.854
1	216	285			190	192		
2	98	148			86	84		
ASA score			5.079	0.079			2.225	0.329
I	79	86			71	63		
II	182	250			153	170		
III	53	97			52	43		
BMI			0.014	0.907			0.282	0.595
≤25	252	346			218	223		
>25	62	87			58	53		
Tumor differentiation			23.860	<0.001			5.455	0.065
I	52	107			52	41		
II	168	258			149	176		
III	94	68			75	59		
Pathological stage			0.155	0.694			0.212	0.645
I+II	103	148			88	83		
III	211	285			188	193		
Surgical approach			15.56	<0.001			0.000	1.000
Laparoscopic surgery	166	166			142	142		
Open surgery	148	267			134	134		

*EOF, early oral feeding; TOF, traditional oral feeding; P1, represents the variable comparison of baseline clinicopathological characteristics before matching; P2, represents the variable comparison of baseline clinicopathological characteristics after matching*.

### Postoperative Complications

Table 2 presents the incidence of each complication in the two groups. In the two matched groups, 43 (15.58%) and 50 (18.12%) patients developed postoperative complications in the EOF and TOF groups, respectively. Although the incidence of postoperative complications was higher in the TOF group, the difference was not statistically significant (*P* > 0.050, Table 2). Serious complications (Clavien-Dindo grade >III) developed in 27.91% (12/43) and 36.00% (18/50) of patients in the EOF and TOF groups, respectively. Reoperation were performed in 11 (3.99%) patients in EOF group and 17 (6.16%) patients in TOF group, and the re-hospitalization rate was 0.36% both in the EOF and TOF groups. The reoperation rate (*P* = 0.245), re-hospitalization rate (*P* = 1.000), and serious complications (Clavien-Dindo grade >III) rate (*P* = 0.405) were not statistically different between the two groups. No 30 day-mortality occurred in either of the groups.

**Table 2:** Comparison of postoperative complications between the EOF and TOF groups after propensity score matching.

**Complications**	**EOF**	**TOF**	**χ^**2**^**	***P*-value**
	**group (*n* = 276)**	**group (*n* = 276)**		
All postoperative complications	43 (15.58%)	50 (18.12%)	0.634	0.426
Clavien-dindo grade >III	12/43 (27.91%)	18/50 (36.00%)		
Anastomosis leakage	7 (2.54%)	8 (2.90%)	0.069	0.793
Duodenal stump leakage	3 (1.09%)	6 (2.17%)	1.017	0.313
Pancreatic fistula	0	0		
Abdominal bleeding	2 (0.72%)	4 (1.45%)	0.674	0.412
Abdominal infection	5 (1.81%)	9 (3.26%)	1.173	0.279
Pulmonary infection	23 (8.33%)	27 (9.78%)	0.352	0.553
Wound infection	5 (1.81%)	4 (1.45%)	0.113	0.737
Wound dehiscence	2 (0.72%)	6 (2.17%)	2.029	0.154
Ileus	6 (2.17%)	6 (2.17%)	0.000	1.000
Reoperation	11 (3.99%)	17 (6.16%)	1.354	0.245
Rehospitalization	1 (0.36%)	1 (0.36%)	0.000	1.000
30-day mortality rate	0	0		

### Tolerance to Oral Feeding

After the propensity score matching, 15 (5.43%) and nine (3.26%) patients had nausea or vomiting in the EOF and TOF groups, respectively (*P* = 0.210). Furthermore, 20 patients (7.25%) in the EOF group and 10 patients (3.62%) in the TOF group had abdominal distention (*P* = 0.060). The tolerance of oral feeding in the EOF and TOF groups was 88.41 and 93.12%, respectively (*P* = 0.056, Table 3).

**Table 3:** Comparison of tolerance to oral feeding between the EOF and TOF groups after propensity score matching.

**Symptoms**	**EOF group**	**TOF group**	**χ^**2**^**	***P*-value**
	**(*n* = 276)**	**(*n* = 276)**		
Nausea or vomiting	15 (5.43%)	9 (3.26%)	1.568	0.210
Abdominal distention	20 (7.25%)	10 (3.62%)	3.525	0.060
Tolerance of oral feeding	244 (88.41%)	257 (93.12%)	3.651	0.056

### Changes in Perioperative Nutritional Markers

In the present study, serum albumin levels and serum prealbumin were used as the nutritional markers. There was no statistical difference between these nutritional markers in the EOF and TOF groups, before and after the surgery (Table 4). The two-way repeated-measures ANOVA analysis revealed that the changes in serum albumin levels from the day before surgery to POD3 was similar between these two groups (*P* = 0.638).

**Table 4:** Comparison of perioperative nutritional markers between the EOF and TOF groups after propensity score matching.

**Nutritional markers**	**EOF group**	**TOF group**	***t*-value**	***P*-value**
Serum preoperative albumin (g/L)	39.27 ± 2.34	39.20 ± 2.24	0.391	0.696
Serum preoperative prealbumin (g/L)	30.89 ± 2.96	30.86 ± 3.06	0.155	0.877
Serum albumin on POD1 (g/L)	34.33 ± 2.35	34.03 ± 2.84	1.355	0.176
Serum prealbumin on POD1 (g/L)	38.51 ± 2.21	28.54 ± 2.32	0.188	0.851
Serum albumin on POD3 (g/L)	31.80 ± 3.17	31.78 ± 2.24	0.080	0.937
Serum prealbumin on POD3 (g/L)	30.08 ± 3.64	30.57 ± 3.45	1.620	0.106

Serum pre-albumin, which has a short half-life and is more sensitive to changes in nutritional status, was also tested, and no statistical difference was found between the two groups, before and after surgery (before surgery: *t* = 0.155, *P* = 0.877; POD1: *t* = 0.188, *P* = 0.851; POD3: *t* = 1.620, *P* = 0.106). The two-way repeated-measures ANOVA analysis also revealed that the changes in serum prealbumin levels from the day before surgery to POD3 was similar between the two groups (*P* = 0.285).

### Postoperative Recovery Outcomes

There was a significant decrease in the time to first passage of flatus or feces in the EOF group, when compared to the TOF group (47.19 ± 12.00 h vs. 58.19 ± 9.89 h, *P* < 0.0001; Table 5). Furthermore, the length of postoperative hospital stay also significantly decreased in the EOF group (6.84 ± 2.31 days vs. 7.72 ± 2.86 days, *P* < 0.0001; Table 5).

**Table 5:** Comparison of postoperative outcomes between the EOF and TOF groups after propensity score matching.

**Outcomes**	**EOF group**	**TOF group**	***t*-value**	***P*-value**
Time to first passage of flatus or defecation (hr)	47.19 ± 12.00	58.19 ± 9.89	11.750	<0.0001
Length of postoperative hospital stay (d)	6.84 ± 2.31	7.72 ± 2.86	3.984	<0.0001

### Sensitivity Analysis for Propensity Score Matching

Since the propensity score only balances the matched variables between the two groups and does not eliminate potential confounding factors, sensitivity analyses were performed to determine the impact of potential confounding factors that fail to match between the two groups. In the sensitivity analysis, the calculations for gamma values ranged between 1.0 (i.e., no hidden bias) and 6.0, stepping by 0.5. The sensitivity analysis tips over significance at the two-tailed α = 0.05 level somewhere between gamma = 5.0 and gamma = 5.5. A gamma threshold was 5.472 for overall postoperative complications and the tolerance of oral feeding. This suggests that an unobserved covariate would be need to produce more than a 5-fold increase in the odds of overall postoperative complications and the tolerance of oral feeding (Supplementary Tables 1, 2).

## Discussion

In China, there is high incidence of gastric cancer. Approximately 40% of new cases of gastric cancer diagnosed every year, worldwide occur in China (1). Various newer therapies, including targeted therapy and immunotherapy, have been developed to improve the survival outcomes of gastric cancer (22). However, the best treatment option for gastric cancer continues to be surgery despite its associated morbidities and the risk of postoperative mortality. Various advancements in the surgical techniques such as minimally invasive surgeries and perioperative care, have led to substantial improvements in postoperative outcomes. Various studies have shown laparoscopic gastrectomy to be associated with lesser blood loss, reduced postoperative pain, faster recovery, and reduced hospital stay (23–25). A key aspect of perioperative care that has been found to improve short-term outcomes includes the adaptation of the ERAS strategy.

Traditionally, oral feeding is started after the appearance of bowel movements, or passage of flatus or defecation after gastric surgery (26). Early oral intake has been considered dangerous due to the fear of anastomotic leakage caused by the increased intraluminal pressure of the postoperative atonic intestine and poor tolerability of patients (6). This concern is particularly evident after total gastrectomy, because the esophageal-jejunal anastomosis is considered to be more prone to develop anastomotic leakage. However, EOF is an important component of ERAS strategy. In the present study, it was found that EOF after radical total gastrectomy for gastric cancer significantly enhanced the recovery of bowel function (*P* < 0.0001) and decreased the length of hospital stay (*P* < 0.0001) without increasing the risk of postoperative complications and mortality. Although a lower occurrence of postoperative complications was observed in the EOF group, the difference was not statistically significant (*P* > 0.05), which implies that EOF is a safe option after radical total gastrectomy. It was also found that there were no significant differences in serum albumin and prealbumin levels before and after surgery in EOF and TOF groups. Hence, it was considered that EOF not only provides nutritional support, but also accelerates the recovery of gastrointestinal function through food stimulation, thereby reducing surgical complications.

In recent years, various studies have shown that EOF after surgery for gastric cancer is feasible and safe (8, 10, 14, 15, 27, 28). Fukuzawa et al. revealed that EOF can promote anastomotic healing (27). A meta-analysis reported by Willcutts et al. (16) analyzed eight RCTs and seven non-RCTs to compare EOF with TOF, and demonstrated that the mean postoperative hospital stay was significantly shorter in the EOF group, with no significant difference in postoperative complications. Liu et al. (15) reported another meta-analysis of six RCTs on EOF after gastrectomy, and demonstrated that postoperative complications and tolerability of oral feeding were not significantly different, and that EOF was associated with a significantly earlier onset of flatulence and defecation, and shorter postoperative hospital stay. However, in the above-mentioned studies, oral feeding was started on postoperative day two or three, while in the present study, oral intake was started in the EOF group on the first postoperative day.

Tolerability of patients is another important factor that should be considered when adopting EOF. Most patients tolerate immediate postoperative feeding without developing major complications, as reported in several studies (26, 29, 30). In the present study, although the rate of intolerance in the EOF group was higher than that in the conventional feeding group, the difference was not statistically significant. This indicates that EOF remains feasible.

Many studies have demonstrated that EOF is safe, and provides nutritional and immunological benefits with better protein kinetics and preservation of the immune system over TOF (31–33). Furthermore, starting EOF can accelerate wound healing and increase anastomotic strength (34). Animal studies conducted using a rat model revealed that starvation after intestinal anastomosis leads to poor quality of healing, and demonstrated that EOF can increase wound healing and strength in esophageal anastomoses (27, 35, 36).

Studies on early oral nutrition after total gastrectomy are limited. Some early RCTs (7, 37) and retrospective studies (14, 38) have reported the outcomes of EOF after total gastrectomy. Kamei et al. revealed that patients who underwent total gastrectomy for gastric cancer were randomized to receive oral enteral nutrition beginning on post-operative day three (39). However, the present results revealed that the mean time to the first passage of flatus or defecation was 58.19 ± 9.89 h, which means that bowel movements starts by third postoperative day three. Hence, oral feeding initiated on POD3 cannot be regarded as EOF. In a RCT and retrospective study that compared early oral nutrition and conventional diet after total gastrectomy, patients who received EOF exhibited no increase in morbidity and anastomosis-related complications, when compared with patients receiving a conventional diet (40). Jang et al. (41) also reported a retrospective study that used propensity score matching to compare EOF with conventional oral feeding after total gastrectomy in gastric carcinoma patients. However, that study was limited by the fact that the patients in the two groups were treated in different time periods, causing the results to be likely in?uenced by the advances in operative skills and perioperative management with time.

At present, surgery is the only curative treatment for gastric cancer. Although, distal gastrectomy is the most common surgery for gastric cancer, total gastrectomy is performed in selected cases with advanced gastric cancer in order to achieve R0 resection. The long-term survival of gastric cancer continues to remain poor despite R0 resection especially for advanced stages of the disease. Hence, multimodality treatment is very important for improving long-term survival. Apart from surgery, other therapies used to treat gastric cancer includes chemotherapy, radiation therapy, immunotherapy and targeted therapy. Since surgery alone is insufficient for most patients with cT2 or higher tumors, perioperative chemotherapy (category 1; preferred) or preoperative chemoradiation (category 2B) are recommended (42–45). Chemoradiation or systemic therapy are the recommended treatment options for medically fit patients whose locoregional cancer is found to be surgically unresectable (46, 47). Postoperative chemoradiation is recommended for all patients following an R1 or R2 resection. Postoperative chemoradiation is also recommended following an R0 resection in selected patients having pT2N0 tumors with high-risk features (poorly differentiated tumor, lymphovascular invasion, neural invasion, age <50 years, and patients who did not undergo D2 lymph node dissection) (48) and for patients with pT3-pT4, any N or any pT, N+ tumors who received less than a D2 dissection (category 1). Patients with pT3-pT4, any N or any pT, N+ tumors who have undergone primary D2 lymph node dissection should receive postoperative chemotherapy (category 1) (49, 50). Recently, biological therapies such as ramucirumab, trastuzumab have been found to improve overall survival in patients with advanced gastric cancer (51). In locally advanced or unresectable cases of gastric cancer, neoadjuvant therapy has been found to be effective in downstaging the tumor (52). In patients showing favorable response to neoadjuvant therapy, subtotal or distal gastrectomy with lymph node dissection can be performed thereby avoiding the morbidities associated with total gastrectomy. Malignant gastric lymphoma refers to a malignant tumor originating from the submucosal lymphoid tissue of the stomach, and may also be a part of systemic malignant lymphoma. Gastric lymphoma is highly sensitive to chemotherapy and can achieve good survival in most patients. Surgery is considered in patients with local complications such as bleeding, obstruction, etc. In addition, the surgical strategy for gastric cancer and gastric lymphoma is different. In gastric adenocarcinoma, due to high incidence of lymph node metastasis, D2-lymph node dissection is performed along with gastrectomy. While, in gastric lymphoma lymph node dissection is not required. Moreover, the first-line chemotherapy for gastric cancer is tegafur, gimeracil, and oteracil potassium (S-1) with oxaliplatin, while the chemotherapy for lymphoma is mostly CHOP (cyclophosphamide + doxorubicin + vincristine + prednisone).

There were some limitations of the present study. First, the present study was retrospective in nature. Retrospective studies have their own biases, which may not be correctable even with propensity score matching. Although propensity score matching can balance the confounding factors between the two groups, the one-on-one matching itself will result in a decrease in the sample size of the pairing and decrease the statistical efficiency to some extent. Furthermore, only observed confounders could be included in the construction of the propensity scores which does not represent all confounding factors. Some other potential confounders not included in this study may affect oral feeding after surgery, for example, anesthetics. The use of opioid analgesics may cause nausea and vomiting in some patients after surgery. This will affect the patient's early oral intake. In addition, the amount of oral intake in the early postoperative period is also a potential confounding factor. The difference in early oral intake of different patients will have certain effects on the tolerance of enteral nutrition and nutritional indicators. Therefore, we intend to include these factors in our future research. Second, this was a single-center study. A single hospital-based design might limit the generalizability of this study. Third, the sample size of this study was relatively small. For safety evaluation, future studies with larger sample size are required.

## Conclusion

The present study reveals that EOF may be safe and feasible after radical total gastrectomy, with faster postoperative recovery and no increased risk of postoperative complications. Future prospective multicenter studies are required to validate the findings of the present study. A wider clinical use of the ERAS strategy can help in significantly reducing hospital cost and improving the postoperative outcomes of patients with gastric cancer.

## Data Availability Statement

The datasets used in this study are available from the corresponding author upon reasonable request.

## Ethics Statement

All the procedures performed in this study that involved humans were in accordance with the ethical standards of the Research Ethics Committee of the Fourth Military Medical University.

## Author Contributions

GJ and JW conceived and designed the study. JW wrote the paper. MY participated in the statistical analysis. QW reviewed and edited the manuscript. All authors read and approved the manuscript and agree to be accountable for all aspects of the research in ensuring that the accuracy or integrity of any part of the work are appropriately investigated and resolved.

### Conflict of Interest

The authors declare that the research was conducted in the absence of any commercial or financial relationships that could be construed as a potential conflict of interest.
